# American Dental Convention

**Published:** 1858-08

**Authors:** 


### FOURTH ANNUAL SESSION OF THE AMERICAN DENTAL CONVENTION.

It will be impossible to give in the limited pages of The Dental Reporter, a full account of this gathering of Dentists, from all parts of the nation, and as most of our readers are also (or should be) readers of the Dental Register of the West, we must ask them to wait patiently the report as it will appear in that valuable quarterly, while we give a more brief account of the convention.

This Fourth Annual Convention was opened at the Melodeon

Hall, on the 3d of August, when the following gentlemen were in attendance :

G. H. Perine, New York City ; John Allen, New York City ; B. Wood, Nashville, Tenn. ; L. W. Sutton, Greenport, Long Island, N. Y.; John J. Blecher, Findlay, Ohio; J. R. Williams, Monongahala City, Pa.; W. H. Morgan, Nashville, Tenn.; Dr. Johnston, Staunton Va.; W. M. Wright, Pittsburg, Pa, J Westbay, Pittsburg, Pa.; R. Vandervort, Pittsburg, Pa.; W. B. Roberts, New York City ; W. H. Atkinson, Cleveland, O.; A. Blake, Sardina, N. Y.; P. Rogers, Shelbyville, Ky.; Martin Tefft, West Poulteny; W. B. Ingersoll, Oberlin, O.; D. R. Greenlee, Meadville, Pa.; W. Kingsly, Freeport, Ill.; Corydon Palmer, Warren, O.; S. M. Goode, Madison, Ind.; A. Leslie, St. Louis, Mo.; Charles Abbey, Philadelphia, Pa.; S. F. Dawes, Louisville, Ky.; —— Stone, Lexington, Ky.; Isaiah Forbes, St. Louis, Mo.; W. A. Cox, Wheeling, Va.; Rogers, Utica, N. Y.; M. Decamp, Mansfield, O.; Frank Fuller, Portsmouth, N. H.; A. M. Assay, Philadelphia, Pa.; J. W. Grant, Lancaster, Ky.; H. McCellum, Augusta, Ky.; J. F. Wilson, Louisville, Ky.; A. L. Dwyer, Louisville, Ky.; A. E. Lyman, Newton Falls, O.; C. M. Kelsey, Mount Vernon, O.; J. Murray, Beaver, Pa.; E. C. Sloan, Irontown, O.; H. J. McKellops, St. Louis, Mo.; S. Dunham, St. Louis, Mo.; W. S. Wilkinson, Philadelphia, Pa.; Thomas L. Vanderbeck, Philadelphia, Pa.; G. W. Barrere, Hillsboro', O.; R. McDowell, Wooster, O.. H. A. Smith, Oxford, O.; J. Mansfield, Niles, Mich.; P. S. Grimes, Kalamazoo, Mich.; R. Peckover, Paris, Ky,; W. P. Hall, Piqua, O.; S. B. Brown, Piqua, O.; Bryan & Cole, Memphis, Tenn.; S. Clippinger, Bellefontaine, O.; J. F. Siddall, Oberlin, O.; Geo. Lupton, Shelbyville, Ind.; Geo. P. Newlin, Danville, Ky.; David Cloud, Greencastle. Ind.; John F. Johnston, Indianapolis, Ind.; W. Harbaugh, Piqua, O.; T. J. Chandler, Rochester Pa.; E. L. Childs, Pittsfield, Mass.; J. W. Fulton, Paris, Ill.; Geo. Watt, Xenia, O.; G. W. Kendall, Newport, Ky.; D. W. Roudebush, Covington, Ky.; Franklin Bell, Greenfield, O.; A. Berry, Raymond, Miss.; W. W. Allport, Chicago, Ill.; A. Terry, Norwalk, O.; H. Jameson, Lyons, N. Y.; E. Conway, Dayton, O.

The Convention was called to order by Dr. Taylor, of Cincinnati. After the reading of the records of the last meeting a committee appointed for that purpose, reported through Dr. Taft, the following

ORDER OF BUSINESS.

1st. President's Address.
2d. Election of Officers.
3d. Reports of committees.
4th. Reading of original papers and essays.

5th. Discussion of the following topics in order: (a) the best means of securing good teeth; (b) the best means of preserving the teeth; (c) the treatment of exposed nerves; (d) mechanical dentistry; (e) exhibition of instruments, &c.; (f) filling teeth.

6th. Miscellaneous business.

A letter from Dr. Tucker, of Boston, the vice president of the Convention was read, regretting and explaining his necessary absence.

The President, Dr. Taylor, then made a brief and pointed address in which he noticed the history, and commented on the education of the profession. The address was eminently acceptable.

The following officers were then elected:

*President*, Dr. ISAIAH FORBES, of St. Louis. *Vice-President*, Dr. ROGERS, of Utica, New York. *Recording Secretary*, Dr. TAFT, of Cincinnati. *Corresponding Secretary*, Dr. D. S. CHASE, of Augusta, Ga. *Treasurer*, Dr. W. B. ROBERTS, of New York.

The Convention then adjourned till 3, P. M.

At their re-assembling, the President elect was conducted to the Chair, and made some appropriate remarks.

On motion of Drs. Wright, of Pittsburg, and Perine of New York, the thanks of the Convention were returned to Dr. Taylor and the other retiring officers.

The committee appointed to audit the Treasurer's accounts, reported a balance in the treasury of $262 96.

The discussion of the topic—*The best means of securing good teeth*—was then opened by the reading of a written essay by Dr. Richardson of this city. The ground taken by Dr. R. was that defective teeth are caused by a lack of proper nutrition, and he suggested whether as the use of certain salts remedied that deficiency of osseous matter which causes rickets, sound teeth might not be promoted by some particular diet.

[This matter will be found ably discussed in the columns of the DENTAL REPORTER, by Prof. St. John, of *The Eclectic College of Medicine*, of this city, whose paper, the commencement of a series, will be found in another part of this number of the Reporter.]

Dr. Atkinson, of Cleveland, thought the best way to secure good milk teeth would be to have healthy children properly circumstanced, and that due attention to the milk teeth would insure perfect permanent teeth.

Dr. Ingersoll, of Oberlin, thought that defective teeth were caused by an imperfect organization, not always external, but frequently occult in its formation, and deceptive in its character, which was the results of depraved appetites, and fashionable customs, for it was not originally intended that men should lose their teeth any more than any other organ.

Dr. Wright, of Pittsburg, traced defective teeth to poor constitutions. He thought they were hereditary.

Dr. Perine, of New York, mentioned the relations between the chemical composition of the teeth and phosphate of lime.

Dr. Rogers, of Utica, did not consider that treatment of this subject, which concerned itself with the selection of persons possessing proper constitutions, practical. Men and women would marry without regard to the laws of Dental physiology.

Dr. Watt's theory of defective teeth, found the cause of their condition in the acids of food, the saliva, hot drinks, &c., rather than in defective constitutions.

Owing to the noise of the street, on which the Melodeon Hall is situated, the Convention adjourned to meet at the lecture room of the Ohio Dental College, at 8 o'clock, on the morning of the 4th.

### SECOND DAY'S PROCEEDINGS.

The Convention met in the Ohio Dental College, at 8 o'clock, August 4th, and the discussion of the first topic—the best means of securing good teeth—was resumed.

Dr. F. Fuller thought that all that could be done was to secure in the mother, as good a constitution and as good health as possible. This should be done, he thought, not by the use of phosphate of lime, but by a healthy regimen. The Irish had, generally, poor teeth. Their diet was chiefly vegetable, and did not contain enough lime.

Dr. Atkinson suggested that brittleness was caused by a deficiency of gellatine, and an excess of earthy matter.

Dr. Roberts believed defective teeth to be, to some extent, hereditary. He was disposed to attribute the defective teeth of Americans, to their mixed nationality. The African degenerated in general constitution by mixture with other races, and possibly the commingling here of English, French, German, &c., affected the condition of the teeth. For if persons of narrow jaws, intermarry with those of wide teeth, might not the children have wide teeth and narrow jaws, and consequently bad and cramped teeth. He did not believe in internal decay; nature, in placing the enamel on teeth had indicated where the danger would come from.

Dr. Taft, of Poultney, Vt., had learned from his brother in Africa, that the natives, when barbarous, had good teeth; when civilized their teeth decayed. He had noticed that on one side of Lake Champlain, black decay prevailed, and on the other side white decay.

Dr. Rehwinkle thought perfect teeth would be found in healthy children. If their teeth were decayed, he would recommend cleanliness of the mouth as a remedy. Prepared chalk was as good an application as anything. An excess of earthy matter rendered the under teeth brittle.

Dr. Kelsey recommended a generous diet for the mother, and cleanliness in the child. He would not have the temporary teeth extracted prematurely.

Dr. Stone, of Lexington recommended friction on the gums of children.

Dr. Roberts had seen happy results from rubbing the gums, and commended the practice.

Dr. Asay commended cleanliness of the mouth and friction of the gums. He had noticed that the Irish teeth had white decay. The neglect of the teeth was a sufficient explanation of their defectiveness. Rubbing the teeth and gums of children had not been successful in the family of Dr. Good, of Madison, Indiana.

Dr. Branch would keep in the temporary teeth as long as possible, and supply the deficiency of osseous salts.

Dr. Chandler believed much to depend on the diet of mother and child. In the South, the field servants had good teeth, and the house servants defective.

Dr. Taylor thought if there was a deficiency of phosphates, they should be supplied in the food; bran should be retained in the bread.

Dr. Rogers, of Cincinnati, would have the care of the teeth begun when the enamel was forming.

Dr. Murray, of Beaver, Pa., believed much injury was done to the enamel, at an early age, by cracking nuts, &c., with the teeth. Being a native of Ireland, he was a little alarmed to hear any say the Irish had bad teeth, for he had, before he left his native land, secured, what he regarded as excellent sets of temporary and permanent teeth.

Dr. DeCamp wished for a more general diffusion of knowledge on the subject of the teeth. He would have girls instructed in physiology. They should not spend their time flirting, but study to become healthy mothers of healthy children.

Dr. McKellops asked if decay was caused by chemical causes, ought there not to be chemical remedies?

Dr. Dunham of St. Louis, mentioned that his own teeth were good, although he was dyspeptic, while the teeth of his brothers and sisters were poor, although their health was vigorous.

Dr. Good favored the practice of destroying the deciduous pulp and leaving the tooth.

Dr. Fuller would educate doctors in the law of dental hygiene, and have the doctors educate the people.

Dr. Allport found his patients and himself benefitted by the publication and distribution of a paper he had written on the subject of the teeth. The patients appreciated the care of the teeth and his operations better.

Dr. Wright would retain the deciduous teeth as long as was possible, and fill them if defective.

Dr. Allen, of New York, advised them to avoid extremes in this matter of filling the deciduous teeth.

Dr. Taft, though for retaining the temporary teeth when practicable, would not allow them to remain if they prevented the protrusion of the permanent teeth. He would only occasionally destroy the deciduous pulp.

*The second topic—The Best Means of Preserving the Teeth*—was then discussed.

Dr. Watt thought decay was induced by acids, which were the result of secretion, excretion, or fermentation of the food.

Dr. Berry stated that in Japan the people had good teeth, and he exhibited a specimen of tooth powder used by the Japanese.

Dr. Van Emmon suggested that galvanic action might have something to do with decay.

Dr. Taft thought the best way to preserve teeth was to have the secretions normal. He recommended more attention to the food of children.

Dr. Taylor, at the request of Dr. Stone, gave his opinion as to the effect of Sugar, &c. Candy he thought injurious, while sugar was wholesome.

As this subject had been involved in the consideration of the first topic, it was not further discussed.

Dr. Taylor, from a committee to whom the subject was referred, reporting in favor of paying $404 20 legal expenses, incurred by Dr. Potter, of Norwalk, while defending a suit brought against him by Levi Gilbert, for alleged infringment of a patent.

It appears that sometime since, Levi Gilbert procured a patent for a certain plate for teeth, known as the central-cavity plate, and then endeavored to procure from dentists something for the use of this plate.

Dr. Potter resisted this claim, having used the plate several years ago.

As the case was that of the whole dental prossession, the Convention determined to take it up as their own.

The resolution introduced by Dr. Taylor, provided for the payment mentioned above, but as the funds were low, $250 was ordered to be paid this year and the balance at some future time. The resolution was passed, as the president said—"unanimously, thank God!"

In the afternoon, the Convention discussed the subject of *Treating exposed nerves*—a subject of deep interest to all practitioners of the dental art, as well as to the public. The discussion was very animated and useful, each seeming willing to give the results of his own observations and reflections in exchange for those of others.

Want of space compels us to omit the details of this afternoon's proceedings, but a full report, doubtless, will be presented by the Secretary.

The Convention adjourned to meet at 8 o'clock in the morning, at the same place.

### THIRD DAY'S PROCEEDINGS.

A large share of the forenoon of the third day was devoted to the discussion of the 3rd subject—*Mechanical Dentistry* In the afternoon, the 4th subject — *Plugging Teeth*—was ably discussed; but our space will not allow us to give even a synopsis.

On motion of Dr. McKellops, the Chair appointed a committee, consisting of Drs. Taylor and Watt, of Cincinnati, Atkinson, of Cleveland, Fuller, of Portsmouth, and Stone, of Lexington, Ky., to coöperate with a committee of the Western Dental Society in petitioning Congress for the appointment of dentists in the army and navy.

It was voted to hold the next annual meeting at Niagara Falls.

During their stay in this city, visiting members of the Convention were entertained by their resident brethren of the Queen City. In the afternoon some ten carriage loads of strangers, members of the Convention, were taken about the beautiful environs of the city, to Spring Grove, Clifton, &c., by Dr. Chas. Bonsall and others composing the Committee of Arrangements.

### THE FESTIVAL.

The evening of the third day, was devoted to a banquet, given by the dentists of Cincinnati and vicinity, to their brethren from abroad, and a number of their invited guests at home. Drs. Charles Bonsall, James T. Irwin and John T. Toland were the Committee of Arrangements. The caterers were Mr.

Henry Alms, and Mr. Nicholas Morris. The table was admirably spread, and there was upon it and on the side tables a superfluity of all that was substantial and delicate that could be provided in our well stocked market. The guests marched into the hall, two abreast, escorted by a band of music, and filed down along the tables, when Dr. Taylor happily remarked that he would not spoil a good supper by making a bad speech, and they all fell to work with the zeal of well appetized gentlemen, happy in the possession of good dental operators, natural and artificial.

After a plentiful supply of the abundance of the good things the table afforded had been disposed of, Dr. Taylor arose and read the first regular toast.

TOASTS AND RESPONSES.

1. The American Dental Convention.

Dr. Forbes of St. Louis, the newly elected President of the Convention, was called upon to respond. The Doctor is a ministerial looking individual, with an evident appreciation of men and things. He commenced by speaking of the Convention in a tone which reminded us of a 4th of July orator, when he suddenly paused and inquired if there was a reporter present. There being several in the hall, but all too modest to announce themselves, the orator proceeded, in uncertainty whether his speech would be reported or not. We were astonished to see by the morning papers that it was *not.* He spoke of white haired men with undyed whiskers, the spirit land and Andrew Jackson Davis, and wound up with the assertion that in the other world there would be no gnashing of teeth, and Othello's (the dentist's) occupation would be gone.

2. The Medical profession—Ever zealous in the cause of science, we welcome this night to our festive board, and as fellow soldier in the great battle with disease, we extend them a cordial greeting.

Dr. L. M. Lawson responded in complimentary terms, encouraging dentists to persevere in their profession, and in allusion to his own, put in a claim that the medical profession was the first in order of precedence of all others, on the ground that the abstraction of the rib from Adam's side was a surgical operation. But while some might consider the remark somewhat wanting in reverence, the profession should remenber that men *sometimes* cut their eye-teeth previous to marriage, and in the operation of teething, may have required the aid of a dentist. In addition to the above and other sayings, equally wise, the Doctor said : Medical savans are subdivided into three grand divisions—the first " allopathic, which, according to high authority, is good for men; secondly, homœopathic, which is good for the women and children; and thirdly, the eclectic, which is good for horses and cows." The Doctor doubtless wanted to give the swill-milk eclectic doctors a sly dig in the ribs, but the attempt to change the festive hall into a Gladiatorial show, in which to give his individual spite an airing, was entirely out of place, and was universally condemed by those whose guest he was.

3. Our Brethren of the East.

Response by Dr. John Allen, of New York, formerly of this city. Dr. A. said when he first came here (he didn't say when) there were but two dentists in the city—Dr. Rogers and Dr. Somerville—and now there are fifty, and upon this head he made his speech.

4. Our Brethren of the South.

Response by Dr. D. Chase, of Augusta, Ga.

5th. Our brethren of the North.

Responses by Dr. Atkinson, of Cleveland, and Dr. Allport, of Chicago.

6th. Our brethren of the West.

7. The land of Dudley—though represented by a *Stone*—it proved to be wrought in a highly finished Corinthian Capital.

Response by Dr. Stone, of Lexington, Ky., who gave a brief history of his experience as a traveling tooth extractor.

8. The Granite State—May it always continue to be Fuller represented in all times to come.

This called out Dr. Fuller, of Portsmouth, N. H., who paid a high compliment to the Queen City, and gave as a sentiment, "The Ohio Dental College of Surgeons," which was reponded to by Dr. Taft, of Cincinnati, who spoke of the unity of the dental profession, it was one and not divided like others. He also gave interesting reminiscences of the past.

9. The Medical Profession—Always aiming to be *right*.

Response by Dr. Wright, of Cincinnati. The Doctor thought he used to be a tolerable good dentist himself. He never broke more than one jaw out of a half a dozen, and now he supposed regular dentists didn't break more than one out of every dozen with their improved instruments. He thought there should have been no distinction made in the toasts between the North, South, East, and West. They all belong to one grand union, and to the great American people.

10. The Keystone of the Union—may it always prove the *right* keystone.

This called out Dr. Wright, of Pittsburg, Pa.

11. Our Dental Institutions—No pent up Utica contracts our powers.

Dr. Rogers, of Utica, N. Y. responded.

12. Our Grinding Organs—May they never be found wanting while we have a *white* man to create them.

Response by Dr. White, of Philadelphia.

13. Our Dental Institutions.

14. Our Dental Periodicals.

Responses were made to each of the above.

The pleasures of the evening were enhanced by frequent music from Menter's famous American Coronet Band; and aside from the little splenetic episode of Dr. Lawson, which is alike condemned by physicians and dentists, "all went as merry as a marriage bell," and at the proper time the festivities were brought to a close.

It is but an act of simple justice to Mr. Morris to say of the many excellent things furnished by him, that what was left after the feast, (and there were more than seven baskets-full,) he sent to the Orphan Asylum of Elm street, to the Catholic Orphan Asylum, and the Widow's Home, and in addition he sent to the Orphan's Asylum a large quantity of ice-cream and pyramid-cake.

The discussions of the convention were resumed on Friday, and at noon of that day it adjourned.

During the session of the Convention and afterwards, *all*, both strangers and citizens, appeared to feel that the time had been spent both profitably and pleasantly, and were fully resolved *to meet again next year.*

The full report of this Convention, as it will be found in the Dental Register of the West, must be a document of extreme interest and value to all in the profession, and to that we refer our readers with confidence.

